# Validation of the Japanese version of the barriers questionnaire II in cancer pain management: a cross-sectional study

**DOI:** 10.1186/s12904-020-00606-0

**Published:** 2020-07-09

**Authors:** Naoki Sakakibara, Hiroko Komatsu, Mikako Takahashi, Hideko Yamauchi, Teruo Yamauchi, Ardith Z. Doorenbos

**Affiliations:** 1 Analysis Section, Center for Cancer Registries, Center for Cancer Control and Information Services, National Cancer Center Japan, 5-1-1 Tsukiji, Chuo-ku, Tokyo, 104-0045 Japan; 2 St. Luke’s International Hospital, 9-1 Akashi-cho, Chuo-ku, Tokyo, 104-8560 Japan; 3 Japanese Red Cross Kyushu International College of Nursing, 1-1 Asty Munakata-City, Fukuoka, 811-4157 Japan; 4 Nursing Department, St. Luke’s International Hospital, 9-1 Akashi-cho, Chuo-ku, Tokyo, 104-8560 Japan; 5 Breast Surgery, St. Luke’s International Hospital, 9-1 Akashi-cho, Chuo-ku, Tokyo, 104-8560 Japan; 6 Medical Oncology, St. Luke’s International hospital, 9-1 Akashi-cho, Chuo-ku, Tokyo, 104-8560 Japan; 7 Department of Biobehavioral Health Science, College of Nursing, University of Illinois at Chicago, Chicago, USA; 8 Palliative Care, University of Illinois Cancer Center, 845 S. Damen Ave., Chicago, Illinois 60612 USA

**Keywords:** Cancer, Pain management, Barriers, Palliative care, Analgesics, Japan

## Abstract

**Background:**

The Barriers Questionnaire II (BQ-II) was developed to assess barriers to effective pain management. In this study, we aimed to assess the reliability and validity of the newly developed Japanese version of the BQ-II (JBQ-II).

**Methods:**

This study used a cross-sectional design. The study was conducted an ambulatory infusion center for cancer in a general hospital in Tokyo, Japan. Participants were 120 Japanese patients with cancer and 21 Japanese health professionals with experience in pain management. Cronbach’s alpha coefficient was used to calculate reliability. Test–retest reliability was assessed with Spearman’s intra-class correlation coefficient (ICC). Construct, criterion-related, and discriminant validity were assessed using information about pain management, daily life, mental health, and subjective health.

**Results:**

The Cronbach’s alpha was 0.90 for the JBQ-II, and all ICCs exceeded 0.70 (*P* < 0.01). Factor analysis showed the JBQ-II had a virtually identical structure to the BQ-II, and path analysis supported the JBQ-II constructs. The JBQ-II was weakly correlated with poor mental state (*r* = 0.36, *P* < 0.01). Patients’ JBQ-II scores were significantly higher than health professionals’ scores.

**Conclusion:**

The JBQ-II is a valid and reliable measure of patient-related barriers to pain management among Japanese adult patients with cancer.

## Background

Pain is a symptom commonly experienced by patients with cancer [1]. However, in Japan, patients’ pain is poorly controlled [2]. Patient-related barriers toward pain management contribute to suboptimal pain relief. These barriers include psychological constructs (e.g., beliefs and values) that affect patients’ pain reporting, requests for pain management, and use of analgesics, thereby leading to inadequate pain control [3–5]. A previous study reported that Japanese patients with cancer are reluctant to report pain and seek analgesic use [2].

The Barriers Questionnaire (BQ) and the Barriers Questionnaire II (BQ-II) were developed to determine patient-related barriers to cancer pain management. These instruments have been translated and used in many countries [6–13]. However, no such tools to assess patient-related barriers to cancer pain management were available in Japan. We developed a Japanese version of the BQ-II (JBQ-II) to identify patient-related barriers to pain management among Japanese patients with cancer. This study aimed to evaluate the reliability and validity of the JBQ-II.

## Methods

### Participants, setting, and recruitment

This study used a cross-sectional design. Approval was obtained from the institutional review boards of Keio University (No. 2012–10) and St. Luke’s International Hospital (No. 12-R066). Participation in this study was voluntary. All participants provided informed consent before participating.

Patients with cancer were recruited from an ambulatory infusion center for cancer in a general hospital in Tokyo, Japan. Eligibility criteria were patients: a) aged 20 years or older; b) able to speak and read Japanese; c) with a diagnosis of metastatic or advanced cancer; (d) with an Eastern Cooperative Oncology Group performance status of 2 or less; and (e) with a pain rating of 1 or higher (on a 0–10 scale) in the previous week. Clinical staff identified eligible patients. An impartial researcher described the study to potential participants and obtained consent.

Health professional participants were recruited from the same hospital. The hospital manager was responsible for recruiting these participants. Inclusion criteria were professionals with a valid license as a physician, nurse, or pharmacist, and at least 3 years of experience in providing pain management. A participation request form and an anonymous questionnaire were sent to identified health professionals who met the inclusion criteria. Health professionals who submitted completed questionnaires were considered to have consented to participate in the study.

### Data collection

Patient participants received a packet of questionnaires that included the JBQ-II, the Japanese version of the Brief Pain Inventory (BPI-J), the six-item Kessler Psychological Distress Scale (K6), and the Subjective Health Scale. Patients completed the questionnaires during a hospital visit and returned them to the researchers. To confirm the test–retest reliability of the JBQ-II, the first 20 patient participants were asked to complete the JBQ-II a second time after a 2-week interval.

An anonymous, self-administered questionnaire was distributed to potential health professional participants. This group was asked to complete the JBQ-II as if they were patients with cancer experiencing pain. The health professionals’ questionnaire also covered age, sex, education, length of occupational experience, and length of palliative care experience. Health professional participants completed the questionnaires in their own time and returned them to the researchers by hospital post.

### Measures

The questionnaire used in this survey was created by combining the following tools.

#### Patients

##### JBQ-II

The BQ-II is a 27-item instrument that measures patients’ beliefs about cancer pain and use of analgesics [4]. The BQ-II was developed as an updated version of the BQ [3], based on changes in pain management practices, developments in the literature, and feedback from patients across multiple studies. Participants are asked to rate the extent to which they agree with each statement on a Likert scale from 0 (disagree) to 5 (strongly agree). Total and subscale scores are calculated, with higher scores indicating stronger barriers. The BQ-II comprises four subscales covering physiological effects, communication, harmful effects, and fatalism.

Consent to translate the BQ-II into Japanese was obtained from the original author of the BQ-II. The JBQ-II passed through a process of double back translations, was Japanized, and the content verified by the original author. Finally, clinical and research experts examined the content and validity of the JBQ-II. When the BQ-II was developed, the three-item “disease progression” BQ subscale was removed. However, this subscale was returned when the BQ-II was translated into Japanese, making the JBQ-II a 30-item instrument. Disease progression was considered an item culturally relevant in Japan because of Japanese patients’ attitudes toward cancer pain.

##### BPI-J

The BPI is a reliable and valid scale used to assess pain intensity and its effect on daily living; higher scores indicate interference in daily life. This scale is used worldwide [14–18], including in Japan [19].

##### Pain Management Index (PMI)

The PMI is a scale used worldwide to evaluate pain management [20, 21]. It is based on the World Health Organization pain ladder and BPI pain scores [22]. The scale ranges from − 3 (e.g., a patient in severe pain who has not received an analgesic) to + 3 (e.g., a patient who has taken a strong opioid and is not reporting any pain).

##### K6

The K6 has six questions covering a person’s emotional state, and has been validated in Japan [23, 24]. Each question is scored from 0 (none of the time) to 4 (all of the time). A total score is calculated, with higher scores indicating poor mental health.

##### Subjective health scale

The Subjective Health Scale is a self-assessment of health with four options: “In very good health,” “In fairly good health,” “Not in very good health,” and “Not in good health at all.” The categorization method for this self-assessment of health is frequently used in Japan [25].

#### Health professionals

Health professionals were asked to answer the JBQ-II subjectively, as if they were a patient with cancer experiencing pain. Information was collected on the appropriateness of the JBQ-II as well as on participant characteristics, length of occupational experience, and length of palliative care experience.

### Data analysis

Descriptive statistics for each JBQ-II item were calculated to examine ceiling or floor effects and verify that each item reflected the trend of respondents. Reliability was determined by internal consistency using Cronbach’s alpha coefficient, with test–retest reliability assessed using Spearman’s intra-class correlation coefficient (ICC). Construct, discriminant, and criterion-related validity were also assessed.

Construct validity was evaluated in two different ways to verify whether it was possible to make theoretical predictions about the concept of a barrier. First, we performed exploratory factor analysis using the principal method and promax rotation, which was the method used for the original scale. The fit of the factor model was evaluated based on the results of the screen test, interpretability, and examination of residuals. Although the BQ-II is a 27-item scale, 30 items (including the three disease progression items) were used for the JBQ-II factor analysis.

**Fig. 1:**
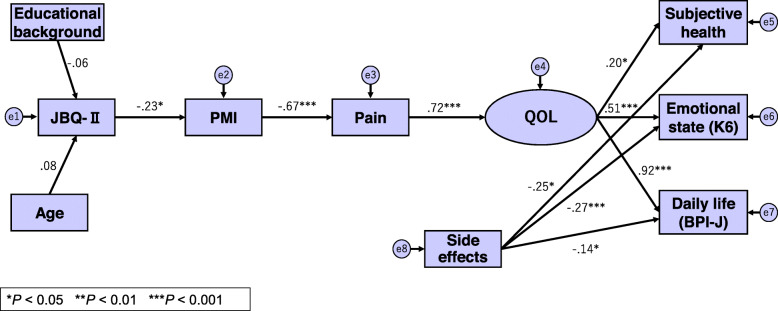
Path diagram for structural equation modeling of the constructive concept. Numerical value:Path coefficient, □:Observation variable, ○:Latent variable, →:Causality, ⓔ:Error. JBQ-II, Japanese Version of the Barriers Questionnaire II (total score, higher scores indicating stronger barriers); PMI, Pain Management Index (−3 to + 3, lower scores indicating inadequate pain management); Pain, pain +/− (dummy variable of 1 for “pain +”); subjective health, healthy/not healthy (dummy variable of 1 for “not healthy”); K6, Six-Item Kessler Psychological Distress Scale (total score, high scores indicate high levels of psychological distress.); BPI-J, Japanese version of the Brief Pain Inventory (total score, higher scores indicate stronger interference in daily life); Side effect, side effect of analgesics +/− (dummy variable of 1 for “side effect −”)

Next, we used path analysis by structural equation modeling to examine a structural model for pain, barriers, and quality of life (QoL), which was developed based on previous studies (Fig. 1) [4, 5, 11]. Barriers to pain management were positioned as psychological constructs (e.g., beliefs and values) toward a threat. This model postulated that strong barriers hinder the use of analgesics as a means of dealing with pain, leading to insufficient pain management. This affects emotional state, daily activities, and subjective health, and QoL declines [4, 5, 11]. We considered subjective health, emotional state (K6), and daily life (BPI) as potential QoL variables, because these factors influence QoL [5, 11]. The path analysis was conducted for 108 patient participants, after excluding 12 who used strong opioids and experienced medium or more severe pain.

The known group method was conducted to explore discriminant validity [4]. Two groups were established: patients were classified as Group A, and a known group of health professionals with special knowledge and training who were serving as patient educators were classified as Group B. The Mann-Whitney U test was used to investigate differences between the two groups’ total scores and the scores for each JBQ-II factor. In theory, Group A scores were expected to be higher than Group B scores.

Regarding criterion-related validity, there were no studies available in Japan to assess associations between the barriers score and outcome measures. Therefore, Pearson’s correlation coefficients for the JBQ-II and K6 domains were calculated. The K6 was chosen as an external index because a barrier is a psychological concept. It was proposed that conceptually-related domains would be weakly correlated with each other. In addition, we asked participants to comment on the feasibility and appropriateness of expression of the JBQ-II. Participants were asked whether the JBQ-II content was clear and easy to understand, whether they found it easy to answer the questionnaire, and how long it took to complete the JBQ-II.

## Results

Questionnaires were distributed to 134 patients, and 123 (91.8%) responded. Valid responses were received from 120 of these respondents (97.5%), after excluding those with missing values or extreme bias. Questionnaires were distributed to 26 health professionals, and 21 (80.8%) responded. Table 1 shows that the patients’ mean age was 56.6 (standard deviation [SD] 11.65) years, and 79.7% were female. The majority of patient participants (73.3%) were well educated. The most frequently identified diagnoses were breast cancer (70.8%), digestive system cancer (18.3%), and urological cancer (9.2%). Most patients (91.7%) had metastatic disease, and more than half had bone metastases.

**Table 1:** Characteristics of patient participants (*N* = 120)

			***N*** = 120
Demographics		*n*	(%)
Age	30–39	7	(5.8)
(56.6 ± *SD* 11.57)	40–49	28	(23.4)
	50–59	33	(27.5)
	60–69	30	(25.0)
	70–89	22	(18.3)
Sex	Male	25	(20.8)
	Female	95	(79.2)
Highest education level	Middle/High school	32	(26.7)
	Vocational school/jr. college	43	(35.8)
	University/graduate school	45	(37.5)
Disease		*n*	(%)
Diagnosis	Breast	85	(70.8)
	Digestive system	22	(18.3)
	Urological	11	(9.2)
	Other	2	(1.7)
Stage	Stage IV	111	(92.5)
	Stage IIIc	6	(5.0)
	Stage IIIb	3	(2.5)
Metastasis	Yes	110	(91.7)
	(Bone metastasis)	67	(57.5)
	No	10	(8.3)
PS ^a^	0	73	(60.8)
	1	40	(33.3)
	2	7	(5.8)
Drugs used	No analgesics used^b^	46	(38.3)
	Non-opioids	57	(46.6)
	Weak opioids	7	(5.8)
	Strong opioids	26	(21.7)

^a^ Eastern Cooperative Oncology Group performance status

^b^ This category includes “Analgesics prescribed but patient has elected not to use,” “Experiencing pain but no analgesics prescribed,” and “Had no pain on the day of the survey”

Health professional participants had a mean age of 33.67 (SD 9.12) years, mean health care experience of 10.81 (SD 9.02) years, and mean palliative care experience of 5.10 (SD 3.18) years. Most of these participants (90.5%) were female.

### JBQ-II score

#### Patients

The internal consistency for the JBQ-II in the patient sample was 0.90. Total JBQ-II scores ranged from 10 to 98 (possible range 0–150), with a mean of 59.18 (SD 21.22). Analyses were performed to determine whether patients’ JBQ-II scores were related to demographic variables, including age, gender, education, marital status, type of cancer diagnosis, and other significant health problems. The only demographic variable that was correlated with total JBQ-II score was a history of previous immunotherapy (*ρ* = − 0.19, *P* < 0.05), which tended to be associated with higher total JBQ-II scores. The JBQ-II total score for patients with previous immunotherapy (*n* = 21) was 64.7 (SD 19.64), whereas that for patients without previous immunotherapy (*n* = 99) was 54.10 (SD 19.95), showing a significant difference between the two groups (*P* = 0.04).

The mean and SD of the individual items of the JBQ-II, floor and ceiling scores, measures of skewness and kurtosis, and I-T correlation data for each item for the total sample are presented in Table 2. The measures of skewness and kurtosis indicated that most items did not show marked deviation from a normal distribution.

**Table 2:** Means, standard deviations, floor and ceiling percentages, skewness, kurtosis, and I-T correlation of the Japanese version of the Barriers Questionnaire-II (total sample)

No	Items	Mean(SD)		Floor (%)	Celling (%)	Skewness	Kurtosis	I-T^a^ correlation
1	Fatalism 1	0.97	(0.94)	38.33	1.67	0.74	0.22	0.27**
2	Harmful Effects: Addiction 1	2.81	(1.42)	7.50	10.00	− 0.43	− 0.66	0.48**
3	Physiological Effects: SE1 (Drowsiness)	2.78	(1.36)	8.33	7.50	−0.49	−0.45	0.32**
4	Harmful Effects: Immune system 1	2.23	(1.51)	21.67	4.17	−0.20	−1.11	0.69**
5	Physiological Effects: SE2 (Confusion)	2.23	(1.46)	19.17	4.17	−0.17	− 0.97	0.71**
6	Physiological Effects: Tolerance 1	3.21	(1.31)	4.17	12.50	−0.81	0.02	0.61**
7	Physiological Effects: Monitor 1	2.64	(1.52)	11.67	11.67	−0.23	−0.90	0.61**
8	Fatalism 2	1.33	(1.28)	28.33	5.00	1.23	1.47	0.11
9	Harmful Effects: Addiction 2	2.55	(1.46)	14.17	6.67	−0.36	−0.76	0.57**
10	Physiological Effects: SE3 (Nausea)	1.98	(1.32)	15.83	3.33	0.16	−0.67	0.44**
11	Communication: Be good 1	1.32	(1.61)	50.00	6.67	0.92	−0.38	0.41**
12	Communiation: Distract MD 1	0.74	(1.18)	62.50	1.67	1.65	2.11	0.48**
13	Harmful Effects: Immune system 2	2.10	(1.52)	23.33	5.00	−0.04	−1.10	0.69**
14	Physiological Effects: SE4 (Embarrassment)	0.79	(1.10)	56.67	0.83	1.31	1.02	0.48**
15	Physiological Effects: Tolerance 2	2.53	(1.48)	12.50	8.33	−0.19	−0.90	0.59**
16	Physiological Effects: Monitor 2	2.37	(1.47)	15.83	6.67	−0.16	− 0.87	0.68**
17	Physiological Effects: SE5 (Constipation)	1.90	(1.48)	25.00	5.83	0.25	−0.81	0.57**
18	Communication: Distract MD 2	0.86	(1.23)	56.67	2.50	1.51	1.81	0.54**
19	Harmful Effects: Immune system 3	1.85	(1.48)	27.50	4.17	0.20	−0.99	0.70**
20	Physiological Effects: SE6 (general item)	1.52	(1.49)	35.83	5.00	0.64	−0.61	0.43**
21	Physiological Effects: Tolerance 3	1.76	(1.50)	25.83	5.00	0.47	−0.84	0.72**
22	Physiological Effects: Monitor 3	2.36	(1.58)	17.50	10.00	−0.04	−1.06	0.78**
23	Harmful Effects: Addiction 3	2.54	(1.51)	12.50	9.17	−0.19	− 0.94	0.74**
24	Fatalism 3	1.05	(1.15)	35.83	3.33	1.62	3.15	0.14
25	Communication: Be good 2	0.91	(1.27)	57.50	0.83	1.20	0.31	0.53**
26	Communication: Distract MD 3	0.68	(1.04)	60.83	0.83	1.74	3.00	0.51**
27	Communication: Be good 3	1.21	(1.45)	46.67	2.50	1.00	−0.16	0.32**
28	Disease Progression 1	3.30	(1.26)	3.33	18.33	−0.64	0.15	0.25**
29	Disease Progression 2	3.40	(1.18)	1.67	20.00	−0.55	0.11	0.36**
30	Disease Progression 3	3.24	(1.15)	0.83	15.00	−0.32	−0.27	0.37**

^a^Item-total correlation **P < 0.01

#### Health professionals

The internal consistency for the JBQ-II total score for the health professional sample was 0.90. The mean total JBQ-II score in these participants was 22.1 (SD 14.79), with total scores ranging from 0 to 51. There were no associations between health professionals’ demographic variables (age, gender, education, years in health care, and years in palliative care) and total JBQ-II scores.

### Reliability verification

#### Internal consistency

Cronbach’s alpha coefficients were 0.90 for the total scale, 0.89 for physiological effects (Factor I), 0.78 for communication (Factor II), 0.86 for harmful effects (Factor III), 0.92 for disease progression (Factor IV), and 0.73 for fatalism (Factor V) (Table 3).

**Table 3:** Factor analysis of the Japanese version of the Barriers Questionnaire-II completed by patients (main factor method: promax rotated factor pattern) and confidence coefficients

(***N*** = 120)						
Factor and question item coefficient	Confidence	Factor loading				
	Entire scale α = 0.90	I	II	III	IV	V
**Factor I: Physiological Effects**	**α = 0.89**					
7. Physiological Effects: Monitor 1		**.834**	−.017	−.074	−.114	.039
15. Physiological Effects: Tolerance 2		**.784**	−.060	−.234	.160	.248
2. Harmful Effects: Addiction 1		**.649**	−.046	.091	−.064	−.278
16. Physiological Effects: Monitor 2		**.624**	.054	.062	.040	.080
9. Harmful Effects: Addiction 2		**.604**	−.015	.080	.007	−.064
6. Physiological Effects: Tolerance 1		**.592**	−.146	.203	.025	.187
21. Physiological Effects: Tolerance 3		**.523**	.261	−.011	.116	.117
23. Harmful Effects: Addiction 3		**.511**	.127	.104	.259	−.016
3. Physiological Effects: SE1 (Drowsiness)		**.482**	−.089	.069	−.166	−.145
10. Physiological Effects: SE3 (Nausea)		**.430**	.014	.121	−.165	−.015
22. Physiological Effects: Monitor 3		**.401**	.247	.163	.177	.047
**Factor II: Communication**	**α = 0.78**					
26. Communication: Distract MD 3		−.198	**.943**	.001	−.020	.018
12. Communication: Distract MD 1		.257	**.680**	−.193	−.125	−.071
11. Communication: Be good 1		.012	**.654**	−.021	−.155	−.084
20. Physiological Effects: SE6 (general item)		.159	**.561**	−.029	−.087	−.288
27. Communication: Be good 3		−.354	**.524**	.138	.111	.164
25. Communication: Be good 2		−.118	**.508**	.150	.066	.311
18. Communication: Distract MD 2		.238	**.461**	.023	−.038	−.118
**Factor III: Harmful Effects**	**α = 0.86**					
4. Harmful Effects: Immune system 1		.018	−.106	**1.034**	−.103	−.065
13. Harmful Effects: Immune system 2		.109	−.069	**.822**	−.004	−.028
19. Harmful Effects: Immune system 3		.210	−.018	**.614**	.038	.049
17. Physiological Effects: SE5 (Constipation)		.001	.146	**.492**	.010	.079
5. Physiological Effects: SE2 (Confusion)		.221	.180	**.407**	.050	.015
14. Physiological Effects: SE4 (Embarrassment)		.116	.151	**.296**	.042	−.080
**Factor IV: Disease Progression**	**α = 0.92**					
29. Disease Progression 2		−.101	.043	−.032	**.963**	−.089
30. Disease Progression 3		−.012	−.041	−.032	**.939**	−.094
28. Disease Progression 1		.021	−.262	.013	**.863**	−.094
**Factor V: Fatalism**	**α = 0.73**					
1. Fatalism 1		.121	−.014	−.017	−.121	**.716**
8. Fatalism 2		−.049	−.060	−.075	−.016	**.704**
24. Fatalism 3		.041	−.125	.049	−.201	**.657**
Factor correlation		I	II	III	IV	V
I		–	.365	.600	.394	.083
II			–	.539	.238	.365
III				–	.197	.203
IV					–	.101
V						–

#### Test–retest reliability

Table 4 shows the results of the test–retest. A significant correlation was identified after calculating the Spearman rank correlation coefficient (*ρ*) for total JBQ-II scores and each subscale. The coefficient for Factor V was *ρ =* 0.49, but that of all other factors were *ρ >* 0.7, indicating that the results can be replicated.

**Table 4:** Test–retest results for the Japanese version of the Barriers Questionnaire-II

	Test (*n* = 21)		Retest (*n* = 21)		
	Mean	*SD*	Mean	*SD*	*Ρ*
Total JBQ-II scores	50.05	21.86	49.71	21.18	.88**
Factor I	24.90	11.22	23.49	16.37	.72**
Factor II	5.90	5.56	5.86	5.69	.88**
Factor III	9.71	7.87	8.95	7.28	.85**
Factor IV	9.48	3.79	8.61	4.14	.75**
Factor V	2.60	2.36	1.90	1.51	.49*

**P < 0.01 *P < 0.05

### Validation verification

#### Construct validity

The construct validity of the JBQ-II was examined by exploratory factor analysis using the principal method and promax rotation (Table 3). Table 5 shows the differences between the BQ-II and the JBQ-II. The three additional disease progression items were an independent factor, whereas the structure of the other four factors did not differ markedly from the original BQ-II. Seven items from one factor in the BQ-II were moved to a different factor in the JBQ-II (Table 5). There were no major changes to the content.

**Table 5:** Differences between Barriers Questionnaire-II and Japanese version of the Barriers Questionnaire-II factors

	BQ-II	JBQ-II
Item	Factor	Factor
5. Physiological Effects: SE2 (Confusion)	I	III
14. Physiological Effects: SE4 (Embarrassment)	I	III
17. Physiological Effects: SE5 (Constipation)	I	III
20. Physiological Effects: SE6 (general item)	I	II
2. Harmful Effects: Addiction 1	III	I
9. Harmful Effects: Addiction 2	III	I
23. Harmful Effects: Addiction 3	III	I

The cumulative contribution rate up to Factor V was 59.33. As the gradient on the scree plot became smaller after Factor V, it was hypothesized that the JBQ-II had a five-factor structure. Factor analysis (specifying five factors) confirmed this structure (Table 3). Each item had sufficient factor loading (0.40 or more), except for one item (item 14). The ratio explaining the total variance of the 30 JBQ-II items over five factors (before rotation) was 52.29%. Path analysis showed that valid paths that fit the construct could be drawn for all characteristics, excluding education and age (Fig. 1).

#### Discriminant validity

Table 6 shows the results of the known group analysis. The null hypothesis was refuted for all factors and total scores. Compared with health professional participants, patients with cancer had significantly higher barriers to pain management, thus indicating discriminant validity.

**Table 6:** Discriminant validity of the Japanese Barriers Questionnaire-II

Factor	Health professionals (*n* = 21)		Patients (*n* = 120)		
	Mean	*SD*	Mean	*SD*	*P*
I	8.45	7.97	27.52	10.89	< .0001
II	5.45	3.97	7.23	6.19	< .0001
III	1.65	2.01	11.09	6.59	< .001
IV	4.90	3.37	9.94	3.35	< .0001
V	1.65	1.57	3.35	2.73	< .001
Total score	22.10	14.79	59.18	21.22	< .0001

#### Criterion-related validity

The analysis of the JBQ-II and K6 showed a weak relationship between Factor II (communication) and the K6 (*r* = 0.20, *P* < 0.05). Creating two segments for PMI, from − 3 to − 1 (“Group not using analgesics sufficiently”) and from 0 to 3 (“Group using analgesics sufficiently”) and conducting a sub-analysis showed that the total JBQ-II score had weak positive correlations with the K6, indicating a poor emotional state (*r* = 0.36, *P* < 0.01). This demonstrated that patient-related barriers have a weak relationship with emotional state, and indicated the JBQ-II had criterion-related validity.

#### Feasibility

In total, 87 patients (72.5%) reported that the question content was clear and easy to understand, 93 (77.5%) reported that answering the questions was clear, and 85 (70.8%) reported that the questions were clear to understand and answer. The length of time needed to answer the JBQ-II ranged from 2 to 20 min (mean 8.34 ± 4.18), which confirmed the feasibility of the instrument.

## Discussion

The overall results showed that the JBQ-II was a reliable and valid instrument for measuring barriers to pain management in Japanese adult patients with cancer. Regarding reproducibility, Factor IV (disease progression) showed a rather low Spearman’s correlation coefficient of 0.49; however, this result might have been affected by patients’ emotional state, symptoms, or condition. The internal consistency of Factor II: Communication and Factor V: Fatalism was slightly lower. Some of the items may have different characteristics than others, but from a content perspective, it makes sense that they belong to the same category. From a holistic perspective of scale, it is a necessary item for observing barriers.

In the factor analysis, some BQ-II items were moved to a different factor in the JBQ-II. Other countries’ versions also differ somewhat in terms of the number of questions and how the sub-scales are divided, but the content of the subordinate construct is virtually identical, and the barriers are evaluated stably from the same viewpoint worldwide [9–13]. Patients’ perception of barriers may therefore reflect cultural backgrounds. However, the content of the subordinate construct was virtually identical across versions, and the barriers were evaluated from similar perspectives worldwide. The constructive concept of the JBQ-II was also virtually the same as that of versions used in other countries. Therefore, it may be considered appropriate as a newly developed scale that reflects cultural background and can measure barriers to pain management in Japan.

The present findings indicated that harmful occurrences or physiological effects might differ depending on the individual’s habits or experiences. It is plausible that the JBQ-II reflects cultural differences regarding awareness of harmful occurrences or physiological effects. In the original questionnaire, subcategories for harmful occurrences included worries about weakening the immune system and concerns about dependence; in Japan, worries viewed as harmful occurrences included weakening the immune system, constipation, and loss of social function or impact on relationships with other people (e.g., confusion or worry about doing something embarrassing).

The path analysis substantiated causal relationships between the JBQ-II → PMI → QoL (Fig. 1), although the path coefficient was low. This suggested that when barriers are high, PMI is low (i.e., inadequate use of analgesics; patient experiences pain) and QoL is affected. As found in previous research [5], no significant path could be drawn where education and age were concerned. However, the characteristic that many of the Japanese respondents were relatively well educated might have influenced the results.

To test the criterion-related validity, we needed to select an external index because there are no indices for the JBQ-II and outcomes. The results showed a correlation between Factor II (communication) and poor emotional state, which is consistent with previous research [5]. Stratification showed a weak correlation between barriers and K6-related external indices (poor emotional state). As there is no evaluation tool in Japan for criterion-related validity, other assessment criteria need to be developed.

This study demonstrated that the JBQ-II can be applied in daily practice. However, as the present study was conducted in a single institution, similar surveys need to be conducted and verified across other settings to assure the generalizability of the findings. The relationship between patient-related barriers and other factors as well as culturally specific barriers should also be further investigated.

## Limitations

There are several limitations to our study. First, this study was conducted in a single institution in Tokyo Japan. Our results may not be applicable other settings. Similar surveys should be conducted in other institutions and regions. A future challenge is to increase the frequency of use and improve the survey’s practical aspects on an ongoing basis. Second, we considered subjective health, emotional state (K6), and daily life (BPI) as potential QoL variables. Therefore, the results might differ if other definitions or a QoL scale were used. Despite these limitations, we conducted multifaceted evaluations of the JBQ-II. Our findings may be useful to assess pain in clinical settings, as well as for epidemiological studies of cancer pain management. It is necessary to provide care that recognizes the presence of barriers to pain management. In addition, from a global perspective, there are many barriers to pain management in Asian and developing countries, which may differ by culture and ethnicity. We believe validated instruments such as the BQ-II need to be available across various languages to integrate global trends.

## Conclusion

The JBQ-II has construct, criterion-referenced, and discriminant validity, and is a reliable and valid index for assessing barriers to pain management among Japanese adult patients with cancer. Therefore, the JBQ-II may be used routinely to improve pain management in Japan.

## Data Availability

Not applicable.

## Abbreviations

BQ-II: Barriers Questionnaire II
JBQ-II: Japanese version of the Barriers Questionnaire II
ICC: Intra-class correlation coefficient
BPI-J: Japanese version of the Brief Pain Inventory
K6: Six-item Kessler Psychological Distress Scale
PMI: Pain Management Index
QoL: Quality of life

## References

[1] Sheinfeld Gorin S, Krebs P, Badr H, Janke EA, Jim HS, Spring B, Mohr DC, Berendsen MA, Jacobsen PB (2012). Meta-analysis of psychosocial interventions to reduce pain in patients with cancer. J Clin Oncol.

[2] Akiyama M, Takebayashi T, Morita T, Miyashita M, Hirai K, Matoba M, Akizuki N, Shirahige Y, Yamagishi A, Eguchi K (2012). Knowledge, beliefs, and concerns about opioids, palliative care, and homecare of advanced cancer patients: a nationwide survey in Japan. Support Care Cancer.

[3] Ward SE, Goldberg N, Miller-McCauley V, Mueller C, Nolan A, Pawlik-Plank D, Robbins A, Stormoen D, Weissman DE (1993). Patient-related barriers to management of cancer pain. Pain.

[4] Gunnarsdottir S, Donovan HS, Serlin RC, Voge C, Ward S (2002). Patient-related barriers to pain management: the barriers questionnaire II (BQ-II). Pain.

[5] Ward SE, Carlson-Dakes K, Hughes SH, Kwekkeboom KL, Donovan HS (1998). The impact on quality of life of patient-related barriers to pain management. Res Nurs Health.

[6] Saifan A, Bashayreh I, Batiha AM, AbuRuz M (2015). Patient- and family caregiver-related barriers to effective cancer pain control. Pain Manage Nurs.

[7] Valeberg BT, Miaskowski C, Paul SM, Rustoen T. Comparison of oncology Patients' and their family Caregivers' attitudes and concerns toward pain and pain management. Cancer Nurs. 2016;39(4):328-34. 10.1097/NCC.0000000000000319 26632879

[8] Jahn P, Kuss O, Schmidt H, Bauer A, Kitzmantel M, Jordan K, Krasemann S, Landenberger M (2014). Improvement of pain-related self-management for cancer patients through a modular transitional nursing intervention: a cluster-randomized multicenter trial. Pain.

[9] Kwon JH, Oh SY, Chisholm G, Lee JA, Lee JJ, Park KW, Nam SH, Song HH, Lee K, Zang DY (2013). Predictors of high score patient-reported barriers to controlling cancer pain: a preliminary report. Support Care Cancer.

[10] Bagcivan G, Tosun N, Komurcu S, Akbayrak N, Ozet A (2009). Analysis of patient-related barriers in cancer pain management in Turkish patients. J Pain Symptom Manag.

[11] Gunnarsdottir S, Serlin RC, Ward S (2005). Patient-related barriers to pain management: the Icelandic barriers questionnaire II. J Pain Symptom Manag.

[12] Jacobsen R, Moldrup C, Christrup L, Sjogren P, Hansen OB (2009). The Danish barriers questionnaire-II: preliminary validation in cancer pain patients. Pain Pract.

[13] Valeberg BT, Hanestad BR, Klepstad P, Miaskowski C, Moum T, Rustoen T (2009). Cancer patients' barriers to pain management and psychometric properties of the Norwegian version of the barriers questionnaire II. Scand J Caring Sci.

[14] Cleeland CS, Ryan KM (1994). Pain assessment: global use of the brief pain inventory. Ann Acad Med Singap.

[15] Cleeland CS, Nakamura Y, Mendoza TR, Edwards KR, Douglas J, Serlin RC (1996). Dimensions of the impact of cancer pain in a four country sample: new information from multidimensional scaling. Pain.

[16] Philip J, Smith WB, Craft P, Lickiss N (1998). Concurrent validity of the modified Edmonton symptom assessment system with the Rotterdam symptom checklist and the brief pain inventory. Support Care Cancer.

[17] Radbruch L, Loick G, Kiencke P, Lindena G, Sabatowski R, Grond S, Lehmann KA, Cleeland CS (1999). Validation of the German version of the brief pain inventory. J Pain Symptom Manag.

[18] Keller S, Bann CM, Dodd SL, Schein J, Mendoza TR, Cleeland CS (2004). Validity of the brief pain inventory for use in documenting the outcomes of patients with noncancer pain. Clin J Pain.

[19] Uki J, Mendoza T, Cleeland CS, Nakamura Y, Takeda F (1998). A brief cancer pain assessment tool in Japanese: the utility of the Japanese brief pain inventory--BPI-J. J Pain Symptom Manag.

[20] Deandrea S, Montanari M, Moja L, Apolone G (2008). Prevalence of undertreatment in cancer pain. A review of published literature. Ann Oncol.

[21] Greco MT, Roberto A, Corli O, Deandrea S, Bandieri E, Cavuto S, Apolone G (2014). Quality of cancer pain management: an update of a systematic review of undertreatment of patients with cancer. J Clin Oncol.

[22] Cleeland CS, Gonin R, Hatfield AK, Edmonson JH, Blum RH, Stewart JA, Pandya KJ (1994). Pain and its treatment in outpatients with metastatic cancer. N Engl J Med.

[23] Kessler RC, Barker PR, Colpe LJ, Epstein JF, Gfroerer JC, Hiripi E, Howes MJ, Normand SL, Manderscheid RW, Walters EE (2003). Screening for serious mental illness in the general population. Arch Gen Psychiatry.

[24] Furukawa TA, Kessler RC, Slade T, Andrews G (2003). The performance of the K6 and K10 screening scales for psychological distress in the Australian National Survey of mental health and well-being. Psychol Med.

[25] Nakano K, Yabe J, Yasumura S (2006). Health practice and total mortality among middle-aged and elderly residents in Sukagawa, Japan. Nihon Koshu Eisei Zasshi.

